# Correlation between prevalence of dental caries and oral hygiene practices among children in Bhubaneswar city, India

**DOI:** 10.6026/973206300214730

**Published:** 2025-12-15

**Authors:** Dipmalla Sahoo, Priyanka Kalimireddy, Jnana Ranjan Swain, Ujala Patnaik, Sonu Acharya, Susant Mohanty

**Affiliations:** 1 Department of Pediatrics and Preventive Dentistry, Institute of Dental Sciences, Siksha 'O' Anusandhan (Deemed to be) University,Bhubaneswar, Odisha, India

**Keywords:** Oral health, dental caries, oral hygiene practices, children

## Abstract

Oral health is vital for systemic well-being as it supports nutrition, social interaction and confidence. Hence, a cross-sectional study among 4800 children aged 6-3 years in Bhubaneswar assessed the link between dental caries and oral hygiene practices. Higher caries prevalence was seen in the 6-9 years group, with no significant sex differences. Caries was strongly associated with brushing frequency and was higher in children using non-fluoridated toothpaste. Proper brushing with fluoridated toothpaste and additional hygiene aids can reduce dental diseases and improve overall health in children.

## Background:

Good oral health is widely recognized as an essential component of overall health, as the oral-systemic connection underscores the influence of oral conditions on systemic well-being and quality of life [1]. Fluoride, both in community-based delivery and individual use, has been extensively documented as one of the most effective cariostatic agents and its combined application plays a crucial role in reducing the incidence and progression of dental caries [2]. Globally, oral diseases, particularly dental caries, continue to represent a significant public health challenge, with substantial impact on children and adults in both developed and developing countries [3]. The burden of dental caries in preschool and school-aged children is multifactorial, being influenced by dietary habits, oral hygiene practices, parental knowledge and socioeconomic status [4]. In India, studies have highlighted wide variations in caries experience across different regions and populations, demonstrating the value of standardized indices such as DMFT and SiC for reliable comparison and epidemiological assessment [5]. Evidence from Nepal further indicates strong associations between poor oral hygiene behavior and higher caries prevalence among school children, stressing the importance of preventive education and early interventions [6]. Similarly, recent data from Afghanistan report alarmingly high caries prevalence among children aged 7-3 years, reflecting the global and widespread nature of this oral health problem [7]. Therefore, it is of interest to correlate between prevalence of dental caries and oral hygiene practices among children in Bhubaneswar city.

## Methodology:

This cross-sectional study was conducted to assess the prevalence of dental caries and its association with oral hygiene practices among school-going children aged 6-3 years in Bhubaneswar City, Odisha, over a period of one year. Ethical approvals for the study was obtained from the Institutional Ethics Committee of the Institute of Dental Sciences, SOA (Deemed to be University), Bhubaneswar and additional permissions were secured from the District Educational Officer and the respective school authorities. Written informed consent was obtained from parents or guardians prior to the participation of their children, ensuring adherence to ethical guidelines and protection of participants' rights. The study population was stratified into two age groups to account for potential variations in caries prevalence and oral hygiene behaviors: Group I included children aged 6-9 years and Group II included those aged 10-3 years. Inclusion criteria were children of both genders within the specified age range who were healthy and present on the day of examination. Children who were unwilling to participate, fell outside the age range, or had systemic medical conditions that could influence oral health were excluded to maintain the integrity of the sample and minimize confounding variables. A random sampling technique was employed to select participants from government schools across Bhubaneswar, ensuring representativeness and minimizing selection bias. Data collection was conducted on school premises under natural lighting conditions, using standardized dental instruments. Each child was examined by trained dental examiners using a plane mouth mirror and a WHO probe, following the World Health Organization (WHO) criteria for the diagnosis of dental caries. All instruments were sterilized daily to maintain hygiene and prevent cross-contamination. In addition to clinical examination, a validated WHO questionnaire assessing oral hygiene practices was administered. The questionnaire, translated into the local language to enhance comprehension, collected information regarding frequency of toothbrushing, type of toothpaste used (fluoridated or non-fluoridated) and other relevant behavioral factors. Caries status was recorded using the DMFT (Decayed, Missing and Filled Teeth) index for permanent dentition and the dmft index for primary dentition, providing a comprehensive assessment of dental caries prevalence and severity. Collected data were systematically compiled and subjected to statistical analysis using IBM SPSS Statistics version 24.0. Descriptive statistics, including frequencies and percentages, were used to summarize demographic characteristics and oral health parameters. The chi-square test was applied to evaluate associations between categorical variables, such as age, gender, brushing frequency and type of toothpaste, with caries prevalence. Independent t-tests were used to compare mean DMFT scores across different groups. A significance level of p < 0.05 was adopted to determine statistical significance. This methodological approach enabled a robust assessment of the prevalence and determinants of dental caries in a large, diverse school-aged population. The combination of clinical examination and validated questionnaire data allowed for a detailed analysis of both biological and behavioral factors affect oral health. By incorporating random sampling, standardized diagnostic criteria and rigorous statistical analysis, the study ensured reliability and validity of the findings. Additionally, stratification by age groups and consideration of gender differences facilitated a nuanced understanding of caries patterns in different subpopulations, providing valuable insights for the planning and implementation of targeted preventive oral health programs.

## Results:

A total of 4,800 school children were examined in this study, comprising 1,946 children in the 6-9 year age group and 2,854 children in the 10-3 year age group. Of these, 2,465 were males and 2,335 were females, with boys slightly outnumbering girls. The overall prevalence of dental caries, defined as DMFT/dmft > 0, was found to be 69.9%. When analyzing the components of the DMFT index, decayed teeth accounted for the majority (66.9%), followed by missing teeth (3.0%) and filled teeth (7.6%). These findings highlight the predominance of untreated decay in this population (Table 1 - see PDF). Caries prevalence was marginally higher in the younger age group (70.9%) compared to the older children (69.1%); however, the difference was not statistically significant (p = 0.186). This indicates that both younger and older children were similarly affected by dental caries, suggesting that the problem persists consistently across these age groups. Gender-wise comparison revealed no significant differences, with males showing a prevalence of 70.8% and females 68.9% (p = 0.164), further suggesting that caries affects both genders nearly equally (Table 1 - see PDF). A more detailed analysis of oral hygiene practices revealed significant associations with caries status. Children who brushed only once daily exhibited a notably higher prevalence of caries (70.8%) compared to those who brushed twice daily (49.5%). The mean DMFT scores further supported this finding, with once-daily brushers having a mean DMFT of 1.48 ± 1.46; nearly double that of twice-daily brushers, who recorded a mean DMFT of 0.76 ± 0.85. This difference was highly significant (p < 0.001), strongly supporting the protective role of increased brushing frequency against dental caries (Table 2 - see PDF). Similarly, the type of toothpaste used was found to be a decisive factor in caries prevalence. Children who used fluoridated toothpaste demonstrated significantly lower caries prevalence (64.2%) compared to those using non-fluoridated toothpaste, where prevalence reached 99.6%. The contrast in mean DMFT scores was striking: children using fluoridated toothpaste had a mean DMFT of 1.00 ± 0.91, while those using non-fluoridated toothpaste recorded a much higher mean DMFT of 3.74 ± 1.39 (p < 0.001). These results provide strong evidence that fluoride use plays a critical protective role in caries prevention among school children (Table 2 - see PDF). Overall, the findings of this study emphasize that while age and gender do not significantly influence dental caries prevalence, oral hygiene behaviors-particularly tooth brushing frequency and the use of fluoridated toothpaste-have a substantial impact on reducing both prevalence and severity of caries.

## Discussion:

The present study highlights the significant role of oral hygiene practices in determining the prevalence of dental caries among children, reflecting trends observed in similar populations. Structured oral hygiene programs have been shown to substantially improve oral health outcomes. For instance, among institutionalized orphans in Bhubaneswar, Odisha, guided interventions resulted in a significant reduction in caries experience, demonstrating the effectiveness of organized preventive strategies in vulnerable populations [8]. This suggests that children with limited access to routine dental care can benefit greatly from structured oral health programs integrated within institutional or school settings. Despite the implementation of awareness initiatives, high caries prevalence continues to be reported among school-going children in Punjab and Haryana, indicating that knowledge alone may not be sufficient to change behavior [9]. These findings are consistent with studies conducted in Tamil Nadu, where disparities in dental caries prevalence were noted among tribal, suburban and urban school children. The differences point to the influence of cultural, environmental and socioeconomic factors on oral health outcomes, suggesting that targeted interventions should consider local determinants to be effective [10]. These variations emphasize the necessity of tailoring oral health promotion strategies to the specific needs of communities, including consideration of cultural norms, dietary patterns and access to oral healthcare services. Early childhood caries has emerged as a major public health issue in Odisha, with high prevalence rates reported in Bhubaneswar [11]. Research indicates that parental knowledge, feeding practices and home oral hygiene routines play a crucial role in influencing children's oral health [12]. Mixed-method studies further highlight that awareness alone is insufficient; parental involvement and education on proper oral hygiene techniques are essential to mitigate caries risk [12]. Similarly, preschool children in Shimla were found to be highly susceptible to early childhood caries, with diet and oral hygiene practices identified as major contributing factors [13]. These findings underline the need for early interventions, including school-based programs and caregiver education, to instill lifelong oral hygiene habits. Regional studies in India provide further evidence of the multifactorial nature of dental caries. In Kerala, high caries prevalence was associated with poor oral hygiene and excessive sugar consumption, emphasizing the combined role of behavioral and dietary factors in disease progression [14]. In Chennai, children exhibited significant caries experience, which was linked to inadequate brushing frequency, irregular dental visits and low awareness of preventive measures [15]. These patterns are echoed in Assam, where differences between rural and urban populations highlight the impact of accessibility to oral healthcare services on caries prevalence [16]. Collectively, these findings indicate that both individual-level behaviors and systemic factors such as healthcare availability contribute to caries risk. Internationally, studies corroborate the influence of behavioral and socioeconomic factors on dental caries. Research from Spain identified risk factors among adolescents, emphasizing those preventive strategies should be age-specific and contextually relevant [17]. Socioeconomic disparities were evident in Jammu, where children attending private schools exhibited lower caries prevalence compared to their peers in government schools, reflecting the influence of economic status on access to oral hygiene products, dietary choices and dental care services [18]. Similarly, evidence from Haryana confirms that oral hygiene practices, including brushing frequency and fluoride use, are strongly linked with caries prevalence among children aged 12-5 years [19]. In Tamil Nadu, studies from Kancheepuram district revealed regional variability in caries prevalence, driven by socioeconomic and behavioral determinants, further emphasizing the complex interplay of risk factors [20]. In Chidambaram, adolescents aged 13-4 years demonstrated high caries levels, reinforcing the urgent need for early preventive interventions and school-based oral health programs that address both hygiene practices and dietary behaviors [21]. These findings collectively highlight that preventive interventions must be comprehensive, integrating education, behavioral modification and access to fluoride-containing oral hygiene products. Furthermore, the role of parental involvement and school-based programs cannot be overemphasized. Children with caregivers who actively monitor and guide oral hygiene practices are less likely to experience high caries prevalence, while school-based dental programs can provide structured opportunities for oral health education, routine check-ups and timely intervention. Behavioral strategies, including promoting brushing twice daily with fluoridated toothpaste and reducing sugary snack consumption, are fundamental to preventing the onset of dental caries. Additionally, policy-level interventions, such as ensuring access to preventive dental care in both urban and rural schools, are necessary to bridge gaps caused by socioeconomic disparities. In conclusion, the evidence from Odisha and other regions underscores that dental caries in children is influenced by a combination of individual behaviors, parental involvement, socioeconomic status and access to preventive care. Structured oral hygiene programs, early education and community-based interventions are critical to mitigating the burden of dental caries. Emphasizing fluoride use, proper brushing techniques and parental guidance, along with addressing regional and socioeconomic disparities, will be essential in promoting long-term oral and general health in children.

## Conclusion:

Oral hygiene practices are vital in preventing dental caries and maintaining overall health. Proper brushing with fluoridated toothpaste and guided programs can reduce caries incidence and improve oral hygiene. Collective efforts by parents, schools and health authorities are essential to encourage lifelong healthy habits in children.
